# Supplementary material for: Exploring environmental and climate features associated with yellow fever across space and time in the Brazilian Atlantic Forest biome
Source: PLoS One. 2024 Oct 7;19(10):e0308560. doi: 10.1371/journal.pone.0308560 (PMC11458019; doi:10.1371/journal.pone.0308560)
Supplement: S1 Table — —* factors that were not applied in the referenced model; D: dependent variable; I: Independent variable; t0: current year; t-1: previous year. (PDF) [file pone.0308560.s001.pdf]

VARIABLE	SOURCE	CODE	DESCRIPTION	NHP CASE MODEL	HUMAN CASE MODEL
**Confirmed Cases of Yellow Fever**					
Epizootics	openData-SUS	prvpL	1 for presence; 0 for absence	D	I
Human prevalence	openData-SUS	prvhL	$\frac{cases\ number_{t0}}{number\ of\ habitants_{t0}} \times 10000$	--*	D
**Land use and vegetation coverage area (%)**	MapBiomas Brasil				
Pasture and Mosaic Agriculture and Pasture		agrps	$Pasture\ (\%) + Mosaic\ Agriculture\ and\ Pasture(\%)$	I	I
Forest Formation		flof		I	I
Reforastation of Forest Formation		reflf	$(\frac{flof_{t0}}{flof_{t-1}} \times 100) - 100; se\ > 0$	I	I
Deforestation of Forest Formation		dsflf	$(\frac{flof_{t0}}{flof_{t-1}} \times 100) - 100; se\ < 0$	I	I
Wooded Restinga		flop		I	I
Reforestation of Wooded Restinga		reflp	$(\frac{flop_{t0}}{flop_{t-1}} \times 100) - 100; se\ > 0$	I	I

Deforastation of Wooded Restinga		dsflp	$(\frac{flop_{t0}}{flop_{t-1}} \times 100) - 100; se\ < 0$	I	I
Other non-forest formation		othnf		I	I
Perennial Crop		peren	Coffee Crop (%) + Other Perennial Crop (%)	I	I
Temporary Crop		ctemp		I	I
Wetlands and River, lake and ocean		wtrlg	$Wetlands(\%) + River, lake\ and\ ocean\ (\%)$	I	I
Savanna Formation and Grassland		svcmp	Savanna Formation (%) + Grassland (%)	I	I
Urban and Other non-vegetated area		urban	Urban area(%) + Other non-vegetated area (%)	--*	I
**Climatical and Environmental**					
Forest fragmentation index (km^{-1})		frag	$\frac{average\ of\ forest\ fragmentation\ perimeters\ in\ km}{average\ of\ forest\ fragmentation\ area\ in\ km^2}$	I	I
Average Temperature (ºC)	Copernicus Climate Change Services (ERA5)	tmpmd	$\frac{\sum\ monthly\ average\ temperature_{t0}}{12}$	I	I
Temperature Range(ºC)		amplt	$maximum\ monthly\ average\ temperature_{t0} - minimum\ monthly\ average\ temperature_{t0}$	I	I

Average Rainfall (mm/day)	Copernicus Climate Change Services (ERA5)	pluvi	$\frac{\sum monthly\ average\ rainfall_{t0}}{12}$	I	I
Rainfall Range		amplp	$maximum\ monthly\ average\ rainfall_{t0} - minumum\ monthly\ average\ rainfall_{t0}$	I	I
Average Humidity (%)	Copernicus Climate Change Services (ERA5)	umid	$\frac{\sum monthly\ average\ humidity_{t0}}{12}$	I	I
Humidity Range		amplm	$maximum\ monthly\ average\ humidity_{t0} - minimum\ monthly\ average\ humidity_{t0}$	I	I
Altitude (m)	Instituto de Pesquisas e Estudos Florestais	altit		I	I
**Koppen Climate Index**	(Alvares *et al.*, 2013)				
Aw		kaw	1 for presence; 0 for absence		
Cfa		kcfa	1 for presence; 0 for absence		
**Human Vaccination and Coverage Strategy**					
Infant vaccine coverage (%)	Data-SUS - tabnet	cvac	Targeted population (<1 year old)	I	I
**NHP specie presence**	(Culot *et al.*, 2019)				
Allouata sp.		nhpal	1 for presence; 0 for absence		
Callithrix sp.		nhpct	1 for presence; 0 for absence		

--* factors that were not applied in the referred model; D: dependent variable; I: Independent variable; t0: current year; t-1: previous yea
