# Supplementary material for: Exploring environmental and climate features associated with yellow fever across space and time in the Brazilian Atlantic Forest biome
Source: PLoS One. 2024 Oct 7;19(10):e0308560. doi: 10.1371/journal.pone.0308560 (PMC11458019; doi:10.1371/journal.pone.0308560)
Supplement: S2 Table — (PDF) [file pone.0308560.s002.pdf]

Biome	NHPs % (n)	Human % (n)	Total of municipalities
Amazon	3.76 (21)	2.51 (14)	558
Atlantic Forest	11.92 (367)	12.18 (375)	3079
Caatinga	1.49 (18)	0.08 (1)	1208
Cerrado	5.67 (81)	3.57 (51)	1429
Pampa	0 (0)	0 (0)	228
Pantanal	0 (0)	0 (0)	22
