# Supplementary material for: Exploring environmental and climate features associated with yellow fever across space and time in the Brazilian Atlantic Forest biome
Source: PLoS One. 2024 Oct 7;19(10):e0308560. doi: 10.1371/journal.pone.0308560 (PMC11458019; doi:10.1371/journal.pone.0308560)
Supplement: S3 Table — (PDF) [file pone.0308560.s003.pdf]

Variables	agrps	altit	amplm	amplp	amplt	ctemp	cvac	dsflf	dsflp	flof	flop	kaw	kcfa	nhpal	nhpct	othnf	peren	pluvi	prevhL	prvp	reflf	reflp	svcmp	tmpmd	umid	urban	wtrlg
agrps					-0.46	-0.44				-0.31	-0.22	0.27	-0.31					-0.42						0.43			
altit			0.22	0.28			0.3			0.26	0.51	-0.24	-0.24			-0.28	0.41					0.27		-0.52			-0.28
amplm		0.22		0.28		0.47	0.3			-0.38					-0.21	-0.25	0.33								-0.78		
amplp		0.28	0.28				0.22										0.23	0.57						-0.22			
amplt	-0.46					0.37	0.21			0.3	0.43	-0.25	0.58		-0.22			0.59				0.21		-0.69			
ctemp	-0.44		0.47		0.37					-0.33			0.39		-0.26			0.23							-0.35		0.29
cvac		0.3	0.3	0.22	0.21						0.26						0.25								-0.31	-0.28	
dsflf											0.22										-0.83						
dsflp																						-0.37					
flof	-0.31	0.26	-0.38		0.3	-0.33					0.47	-0.29		0.22				0.41			-0.28			-0.59	0.52		-0.28
flop	-0.22	0.51			0.43		0.26	0.22		0.47		-0.27						0.32			-0.31	0.43		-0.61			
kaw	0.27	-0.24			-0.25					-0.29	-0.27		-0.32					-0.27						0.46	-0.34		0.21
kcfa	-0.31	-0.24			0.58	0.39						-0.32						0.38						-0.3			
nhpal										0.22					0.3												
nhpct			-0.21		-0.22	-0.26								0.3													
othnf		-0.28	-0.25																					0.21			
peren		0.41	0.33	0.23			0.25																		-0.43		
pluvi	-0.42			0.57	0.59	0.23				0.41	0.32	-0.27	0.38										-0.24	-0.66	0.35		
prevhL																				0.33							
prvp																			0.33								
reflf								-0.83		-0.28	-0.31													0.31			
reflp		0.27			0.21				-0.37		0.43													-0.27			
svcmp																		-0.24									
tmpmd	0.43	-0.52		-0.22	-0.69					-0.59	-0.61	0.46	-0.3			0.21		-0.66			0.31	-0.27			-0.37		0.23
umid			-0.78			-0.35	-0.31			0.52		-0.34					-0.43	0.35						-0.37			-0.21
urban							-0.28																				
wtrlg		-0.28				0.29				-0.28		0.21												0.23	-0.21		
