# Supplementary material for: Exploring environmental and climate features associated with yellow fever across space and time in the Brazilian Atlantic Forest biome
Source: PLoS One. 2024 Oct 7;19(10):e0308560. doi: 10.1371/journal.pone.0308560 (PMC11458019; doi:10.1371/journal.pone.0308560)
Supplement: S4 Table — (PDF) [file pone.0308560.s004.pdf]

Region	State	NHP	Human
Northeast	Bahia	0	
Southeast	Espírito Santo	1	3
	Minas Gerais	6	23
	Rio de Janeiro	4	6
	São Paulo	19	18
South	Paraná	7	0
	Santa Catarina	3	0
**TOTAL**		**40**	**50**
