# Supplementary figures and images for: Exploring environmental and climate features associated with yellow fever across space and time in the Brazilian Atlantic Forest biome
Source: PLoS One. 2024 Oct 7;19(10):e0308560. doi: 10.1371/journal.pone.0308560 (PMC11458019; doi:10.1371/journal.pone.0308560)

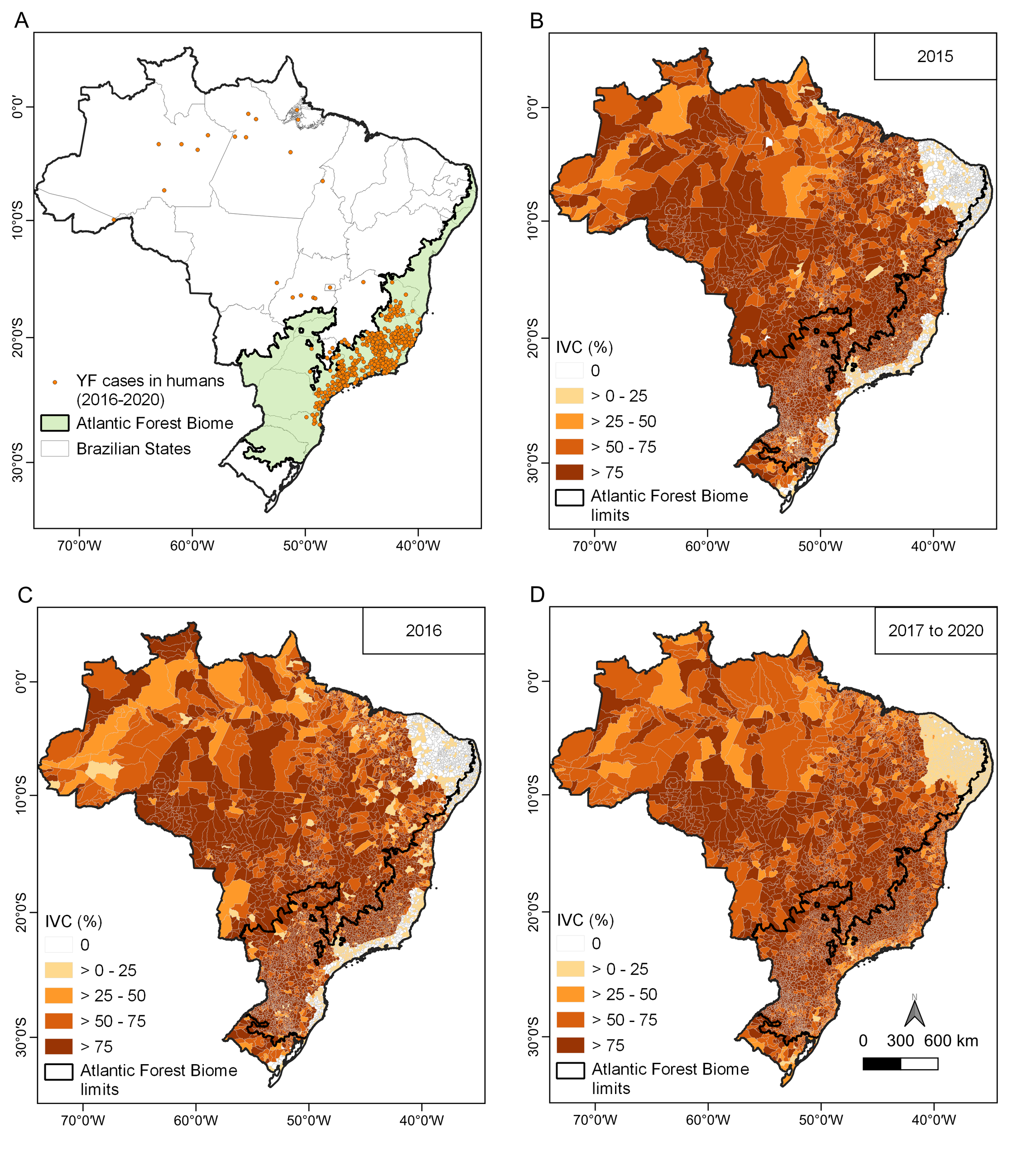

Supplement: S1 Fig — A) Human confirmed cases of YF through 2016–2020, B) Infant Vaccine Coverage in 2015, C) Infant Vaccine Coverage at the beginning of AFB outbreak in 2016, D) Infant Vaccine Coverage during the outbreak in 2017 to 2020. (TIF) [file pone.0308560.s005.tif]

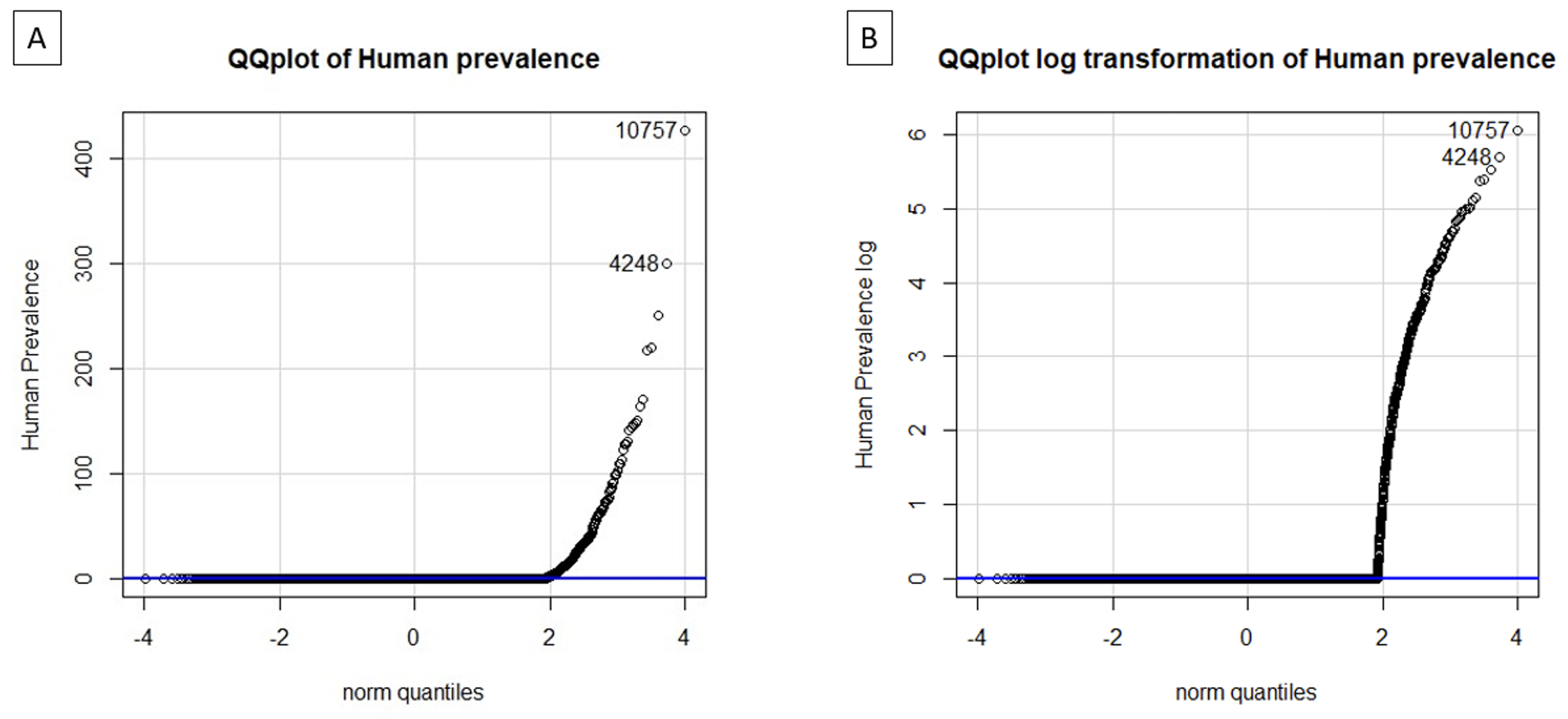

Supplement: S2 Fig — QQ plot depicting the distribution of A) human prevalence; and B) log-transformed human prevalence for normality assessment. (TIF) [file pone.0308560.s006.tif]

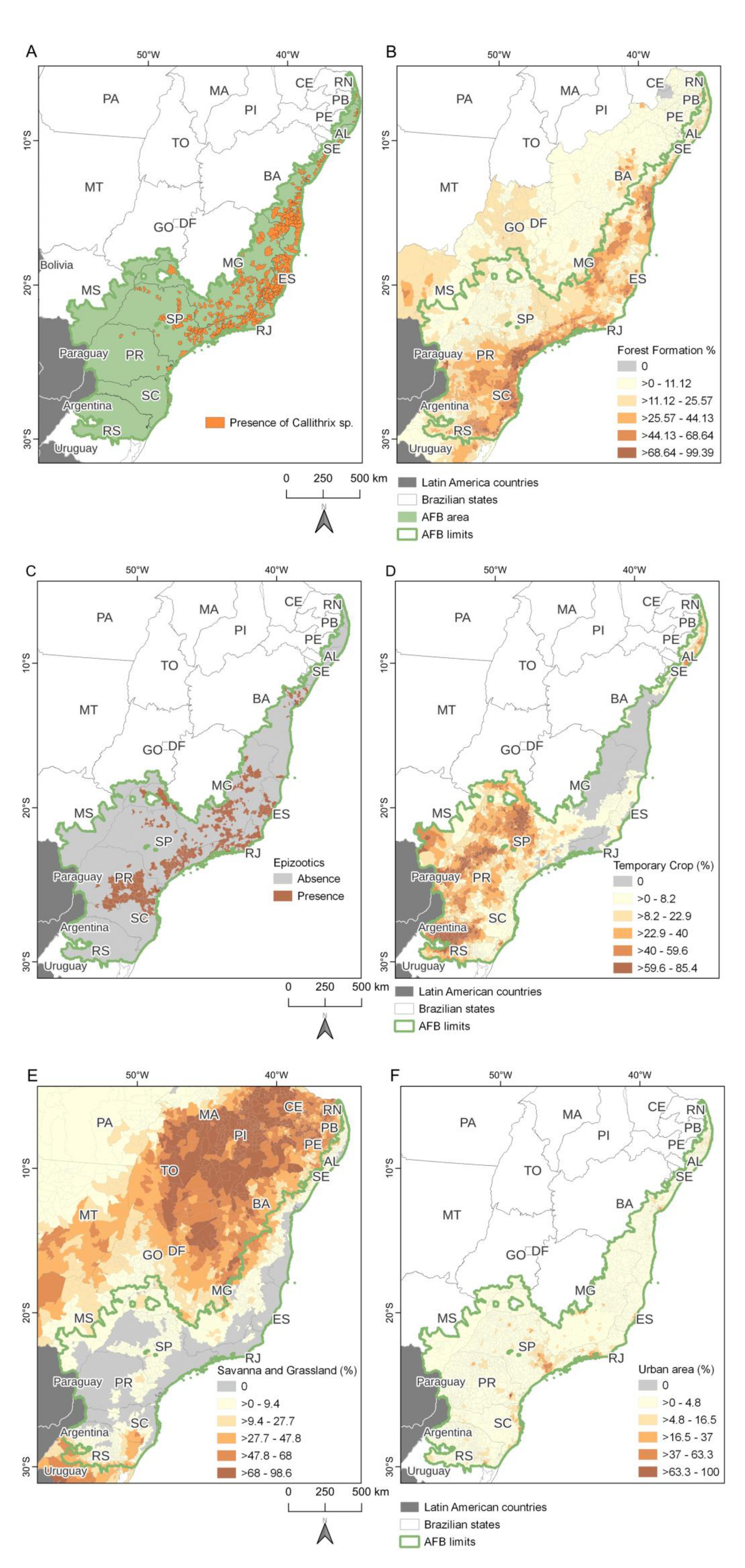

Supplement: S3 Fig — A) Presence of Callithrix sp.; B) Percentage of forest cover in the AFB and in states containing at least one municipality within the AFB; C) Occurrence of epizootics; D) Proportion of land dedicated to temporary crops within the AFB; E) Percentage of savannah and grassland across Brazil; and F) Extent of urban areas within the AFB. Categories graduated by natural breaks. (TIF) [file pone.0308560.s007.tif]

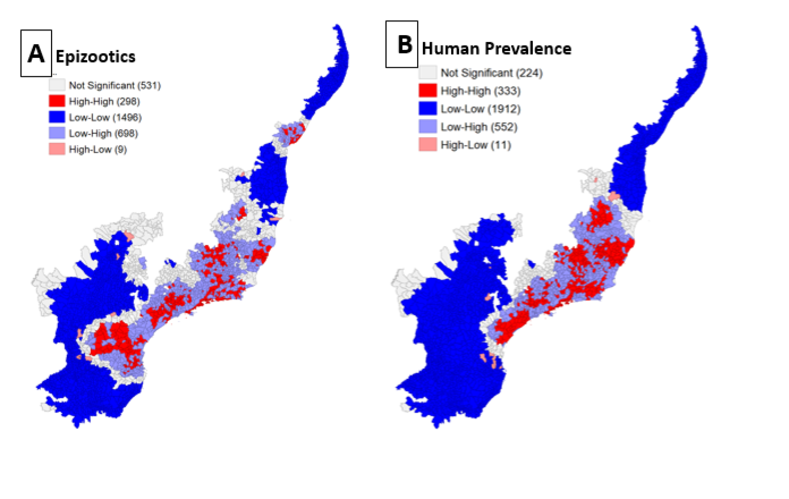

Supplement: S4 Fig — Univariate Local Moran’s I for A) Epizootic Events; and B) Human Prevalence. "High-High" indicates a high intensity of the variable, while "Low-Low" signifies the absence of the variable. (TIF) [file pone.0308560.s008.tif]

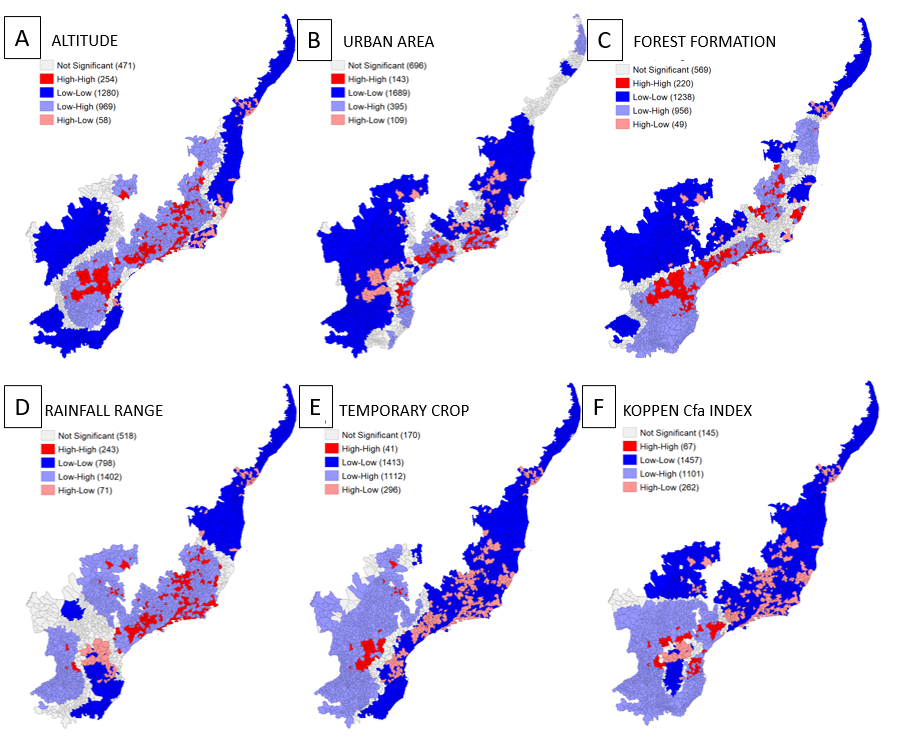

Supplement: S5 Fig — Bivariate Local Moran’s I analysis of epizootics in relation to A) altitude; B) urban areas; C) forest formations; D) rainfall ranges; E) temporary crops; and F) Köppen Cfa index. High-High: high intensity of both variables; Low-Low: absence of both variables; Low-High: high intensity of one variable and absence of the other. The absence of epizootics and a high value of the independent variable; High-Low: The presence of epizootics and a zero or low value of the independent variable. (TIF) [file pone.0308560.s009.tif]

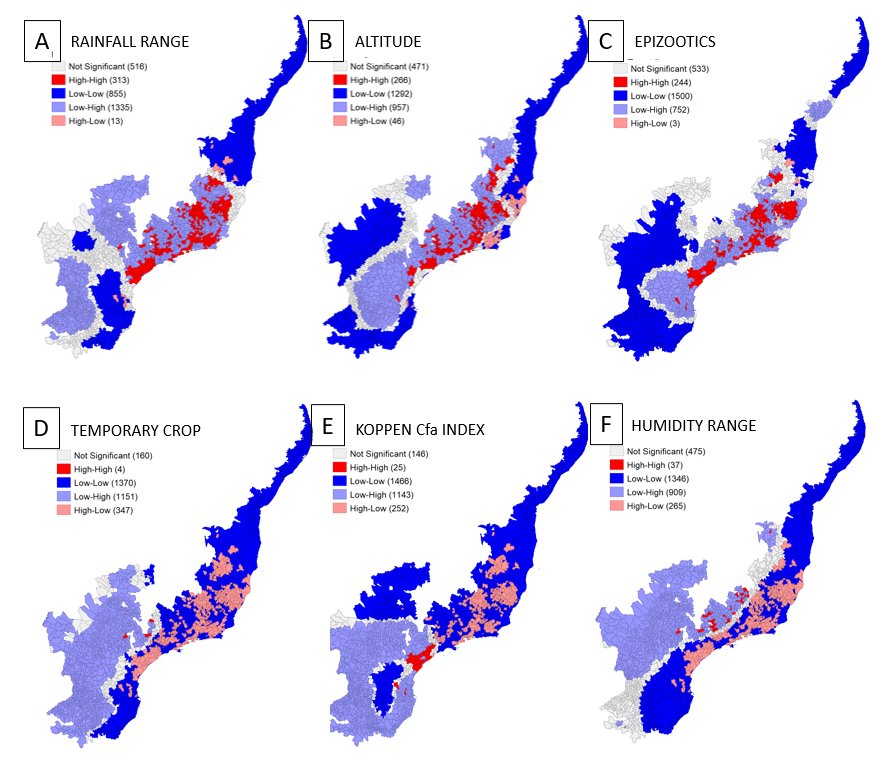

Supplement: S6 Fig — Bivariate Local Moran’s I analysis of human prevalence in relation to A) rainfall variability, B) altitude, C) epizootic events, D) temporary crops, E) Köppen Cfa index, and F) humidity variability. High-High: high intensity of both variables; Low-Low: absence of both variables; Low-High: high intensity of one variable and absence of the other. Low or zero human prevalence and a high value of the independent variable; High-Low: High human prevalence and negligible or zero values of the independent variable. (TIF) [file pone.0308560.s010.tif]
