# Supplementary material for: Exploring environmental and climate features associated with yellow fever across space and time in the Brazilian Atlantic Forest biome
Source: PLoS One. 2024 Oct 7;19(10):e0308560. doi: 10.1371/journal.pone.0308560 (PMC11458019; doi:10.1371/journal.pone.0308560)
Supplement: S1 Appendix — (PDF) [file pone.0308560.s011.pdf]

Exploring environmental and climate features associated with yellow fever across space and time in the Brazilian Atlantic Forest biome

Maíra G. Kersul and Anaiá da P. Sevá

23-jan-2024

Mixed Model for yellow fever

Here we present two models, one based on the variables for human cases and other for non-human-primates (NHPs) cases, both of them set as dependent variables. The independent variables are environmental and climatic, and random variables are time (year) and space (municipalities). The model also considers the distance dependence between municipalities.

Asking for table and fitting it

1. Filtering Atlantic Forest Biome.
2. Transforming variable of human prevalence (prevh), by using log(1+prevh), and adding it in the table.

```
library("readxl")
tab <- read_excel("~/MaiKersul(Dout)/yftab.xlsx")

#Filter to use Atlantic Forest Biome (mtatl)
mtasep <- tab[tab$mtalt>0,]

# Human prevalence transformation
mtasep["prevhL"] <- log(1+mtasep$prevh)

str(mtasep)
```

```
## tibble [15,395 × 42] (S3: tbl_df/tbl/data.frame)
##  $ ano       : num [1:15395] 16 16 16 16 16 16 16 16 16 16 ...
##  $ id6       : num [1:15395] 240120 240140 240220 240260 240325 ...
##  $ x         : num [1:15395] -35.2 -35 -35.1 -35.4 -35.2 ...
##  $ y         : num [1:15395] -6.19 -6.43 -6.4 -5.6 -5.91 ...
##  $ prevh     : num [1:15395] 0 0 0 0 0 0 0 0 0 0 ...
##  $ prevp     : num [1:15395] 0 0 0 0 0 0 0 0 0 0 ...
##  $ tempmed   : num [1:15395] 26.2 26.1 26 26.5 26.3 ...
##  $ amplitemp : num [1:15395] 2.25 2.1 2.24 2.04 2.15 ...
##  $ pluvi     : num [1:15395] 2.24 3.53 2.6 2.63 2.5 ...
##  $ amplipluvi: num [1:15395] 5.09 6.77 5.55 5.42 5.17 ...
##  $ umid      : num [1:15395] 78.6 80.2 79.4 78.3 79.8 ...
##  $ ampliumid : num [1:15395] 10.66 7.75 9.72 9.69 9.48 ...
##  $ altit     : num [1:15395] 66 36 56 46 42 98 28 67 107 29 ...
##  $ amz       : num [1:15395] 0 0 0 0 0 0 0 0 0 0 ...
##  $ caat      : num [1:15395] 0 0 1 89.8 69.7 ...
##  $ cer       : num [1:15395] 0 0 0 0 0 0 0 0 0 0 ...
##  $ mtalt     : num [1:15395] 100 100 99 10.2 30.3 ...
##  $ pamp      : num [1:15395] 0 0 0 0 0 0 0 0 0 0 ...
##  $ pant      : num [1:15395] 0 0 0 0 0 0 0 0 0 0 ...
##  $ agrpst    : num [1:15395] 55.4 36.2 54.5 43.8 19.3 ...
##  $ flof      : num [1:15395] 16.04 16.35 6.77 18.53 23.46 ...
##  $ reflof    : num [1:15395] 0 0.68 0.3 0 0 0 0 0.89 0 0 ...
##  $ desflof   : num [1:15395] 0.68 0 0 6.88 1.72 ...
##  $ flop      : num [1:15395] 0 0 0 0 0 0 0 0 0 0 ...
##  $ reflop    : num [1:15395] 0 0 0 0 0 0 0 0 0 0 ...
##  $ desflop   : num [1:15395] 0 0 0 0 0 0 0 0 0 0 ...
##  $ othnfl    : num [1:15395] 0 0.08 0.03 4.88 6.44 ...
##  $ peren     : num [1:15395] 0 0 0 0 0 0 0 0 0 0 ...
##  $ ctemp     : num [1:15395] 10.14 26.22 18.2 4.98 0.08 ...
##  $ savcamp   : num [1:15395] 6.61 1.04 3.31 23.44 4.6 ...
##  $ urban     : num [1:15395] 1.79 0.53 2.66 1.79 41.27 ...
##  $ vurb      : num [1:15395] 0.562 0 1.527 7.186 1.276 ...
##  $ wetriolg  : num [1:15395] 5.71 7.13 3.96 0.71 2.09 0.66 5.09 1.04 0.2 4.42 ...
##  $ frag      : num [1:15395] 0 0 0 0 0 0 0 0 0 0 ...
##  $ cvac      : num [1:15395] 0 5.74 0 0.08 0.07 0 0.55 0.43 0 0 ...
##  $ vacbi     : num [1:15395] 0 0 0 0 0 0 0 0 0 0 ...
##  $ vacrot    : num [1:15395] 0 0 0 0 0 0 0 0 0 0 ...
##  $ kcfa      : num [1:15395] 0 0 0 0 0 0 0 0 0 0 ...
##  $ kaw       : num [1:15395] 0 0 0 0 0 0 0 0 0 0 ...
##  $ nhpal     : num [1:15395] 0 1 0 0 0 0 0 0 0 0 ...
##  $ nhpctx    : num [1:15395] 0 0 0 0 0 0 0 0 0 0 ...
##  $ prevhL    : num [1:15395] 0 0 0 0 0 0 0 0 0 0 ...
```

Selecting variables to models by evaluating their correlations

Assessed the correlation among variables using the Spearman (ρ) test because of their non-normal distribution.

Were selected independent variables that showed a significant correlation ($p<0.05$) with the dependent variable.

For independent variables with strong correlation between each other (ρ > 0.2 or ρ < -0.2 and p < 0.05) there were selected the one most significant with the dependent.

Correlation tests to select variables for epizootic model

1. Evaluating the correlation between all numerical variables by creating a matrix (excluding columns 1 to 4 and 14 to 19 of the table)
2. Select dependent x independent variables p<0.05

```
# Epizootics correlations
library("psych") #for correlation matrix
tabpnhcorr <- print(corr.test(mtasep[,-c(1:4,14:19)],
                             method="spearman", adjust="none",
                             use="pairwise"),
                   short= FALSE)

# Adjusting the table for analysis
tabpnhcorr["nomevar"] <- row.names(tabpnhcorr)
library("stringr")
col <- str_split_fixed(tabpnhcorr$nomevar, "-", 2)
tabpnhcorr <- cbind(tabpnhcorr,col)
names(tabpnhcorr) [c(8,9)] <- c("var1","var2")
pnhcorr <- tabpnhcorr[,c(2,4,8,9)]
deppnh <- pnhcorr[pnhcorr$var1=="prevp",]
indeppnh <- subset(pnhcorr,var1!="prevp")

# select dependent x independent variables p<0.05 (column raw.p)
depselectpnh <- deppnh[deppnh$raw.p<0.05,]
```

Correlation tests to select variables for human model

1. Evaluating the correlation between all numerical variables by creating a matrix (excluding columns 1 to 4 and 14 to 19 of the table)
2. Select dependent x independent variables p<0.05

```
# Epizootics correlations
library("psych") #for correlation matrix
tabhumcorr <- print(corr.test(mtasep[,-c(1:4,14:19)],
                             method="spearman", adjust="none",
                             use="pairwise"),
                   short= FALSE)

# Adjusting the table for analysis
tabhumcorr["nomevar"] <- row.names(tabhumcorr)
library("stringr")
col <- str_split_fixed(tabhumcorr$nomevar, "-", 2)
tabhumcorr <- cbind(tabhumcorr,col)
names(tabhumcorr) [c(8,9)] <- c("var1","var2")
humcorr <- tabhumcorr[,c(2,4,8,9)]
dephum <- humcorr[humcorr$var1=="prevhL",]
indephum <- subset(humcorr,var1!="prevL")

# select dependent x independent variables p<0.05 (column raw.p)
depselecthum <- dephum[dephum$raw.p<0.05,]
```

Correlation test of independent variables

Comparison between independent variables.

Identifying the independent variables with high correlation among each other (ρ > 0.2 or ρ < -0.2 and p < 0.05) to then identify the ones that have higher correlation with dependent variable.

```
#to select independent variables with p <0.05 correlations with dependent variable
indepselectp <- indeppnh[indeppnh$raw.p<0.05,]
#to select independent variables with rho > 0.2 and rho < -0.2 correlation with independent v
ariables
indepselectr1 <- indepselectp[indepselectp$raw.r>0.2,]
indepselectr2 <- indepselectp[indepselectp$raw.r<(-0.2),]

#to unite their results in unique table
indepselectr <- rbind(indepselectr1,indepselectr2)
```

EPIZOOTICS MIXED MODEL

In this model the variable of presence and absence (binomial) of NON-HUMAN PRIMATE is the dependent.

The selected independent variables were forest (flof), Callithrix sp. (nhpctx), savannah and grassland (savcamp).

The “random” are random variables, as time in year (ano) and municipality code (id6).

```
library("MASS") # for glmmPQL
library("nlme") # for Spatial correlation
fit7 = glmmPQL(prevp ~ flof + nhpctx + savcamp,
               random = ~ 1|ano|id6,
               corr = corSpatial(form=~jitter(x)+y, type = "exponential"),
               family = binomial, data = mtasep)
summary(fit7)
```

```
## Linear mixed-effects model fit by maximum likelihood
##   Data: mtasep
##   AIC BIC logLik
##    NA  NA     NA
##
## Random effects:
##  Formula: ~1 | ano | id6
##  Structure: General positive-definite, Log-Cholesky parametrization
##              StdDev    Corr
## (Intercept) 1.5025867 (Intr)
## 1 | anoTRUE 0.7066709 0.897
## Residual    0.3780122
##
## Correlation Structure: Exponential spatial correlation
##  Formula: ~jitter(x) + y | id6
##  Parameter estimate(s):
##        range
## 3.375438e-06
## Variance function:
##  Structure: fixed weights
##  Formula: ~invwt
## Fixed effects:  prevp ~ flof + nhpctx + savcamp
##                  Value  Std.Error    DF   t-value p-value
## (Intercept) -6.388144 0.12301285 12314 -51.93070  0.0000
## flof         0.032452 0.00323693 12314  10.02559  0.0000
## nhpctx       1.511479 0.15768352  3077   9.58552  0.0000
## savcamp     -0.002641 0.00810039 12314  -0.32601  0.7444
##  Correlation:
##         (Intr) flof   nhpctx
## flof    -0.796
## nhpctx  -0.246 -0.023
## savcamp -0.287  0.137  0.022
##
## Standardized Within-Group Residuals:
##        Min         Q1        Med         Q3        Max
## -4.7882943 -0.1737700 -0.1259626 -0.1103576  6.0283781
##
## Number of Observations: 15395
## Number of Groups: 3079
```

```
library("sjPlot") # for organize table
tab_model(fit7)
```

	prevp		
Predictors	*Odds Ratios*	*CI*	*p*
(Intercept)	0.00	0.00 – 0.00	**<0.001**
flof	1.03	1.03 – 1.04	**<0.001**
nhpctx	4.53	3.33 – 6.18	**<0.001**
savcamp	1.00	0.98 – 1.01	0.744

N id6	3079
Observations	15395

```
library("MuMIn") # for R squared generation
r.squaredGLMM(fit7)
```

```
##                    R2m       R2c
## theoretical 0.07524785 0.6173917
## delta       0.01758554 0.1442854
```

HUMAN MIXED MODEL

In this model the variable of prevalence (log transformed) of HUMAN is the dependent.

The selected independent variables were epizootics presence (prevp), temporary crop (ctemp), savannah and grassland (savcamp) and urban area (urban).

The “random” are random variables, as time in year (ano) and municipality code (id6).

```
fit7h = glmmPQL(prevhL ~ prevp + ctemp + savcamp + urban,
               random = ~ 1|ano|id6,
               corr = corSpatial(form=~jitter(x)+y, type = "exponential"),
               family = poisson, data = mtasep)
summary(fit7h)
```

```
## Linear mixed-effects model fit by maximum likelihood
##   Data: mtasep
##   AIC BIC logLik
##    NA  NA     NA
##
## Random effects:
##  Formula: ~1 | ano | id6
##  Structure: General positive-definite, Log-Cholesky parametrization
##             StdDev       Corr
## (Intercept) 7.421662e-04 (Intr)
## 1 | anoTRUE 2.306445e-11 0
## Residual    1.829446e+00
##
## Correlation Structure: Exponential spatial correlation
##  Formula: ~jitter(x) + y | id6
##  Parameter estimate(s):
##        range
## 2.729358e-06
## Variance function:
##  Structure: fixed weights
##  Formula: ~invwt
## Fixed effects:  prevhL ~ prevp + ctemp + savcamp + urban
##                  Value  Std.Error    DF    t-value p-value
## (Intercept) -2.1933507 0.08231135 12312 -26.647003   0e+00
## prevp        2.7519149 0.12091237 12312  22.759580   0e+00
## ctemp       -0.0797367 0.00838935 12312  -9.504507   0e+00
## savcamp     -0.0329823 0.00947948 12312  -3.479336   5e-04
## urban       -0.0490012 0.01140208 12312  -4.297561   0e+00
##  Correlation:
##         (Intr) prevp  ctemp  savcmp
## prevp   -0.490
## ctemp   -0.389  0.069
## savcamp -0.291  0.066  0.069
## urban   -0.271 -0.144  0.028  0.020
##
## Standardized Within-Group Residuals:
##         Min          Q1         Med           Q3         Max
## -0.72164555 -0.16054196 -0.10100171 -0.04226967 58.69571180
##
## Number of Observations: 15395
## Number of Groups: 3079
```

```
tab_model(fit7h)
```

	prevhL		
Predictors	*Incidence Rate Ratios*	*CI*	*p*
(Intercept)	0.11	0.09 – 0.13	**<0.001**
prevp	15.67	12.37 – 19.86	**<0.001**
ctemp	0.92	0.91 – 0.94	**<0.001**
savcamp	0.97	0.95 – 0.99	**0.001**

urban	0.95	0.93 – 0.97	**<0.001**
N id6	3079		
Observations	15395		

```
r.squaredGLMM(fit7h)
```

```
##                  R2m        R2c
## delta     0.26174000 0.26174005
## lognormal 0.58639784 0.58639794
## trigamma  0.03599605 0.03599605
```
